# Seismic Isolation Performance of Seismic Metamaterials Based on Embedded Dual-Resonator Coupled Auxetic Materials

**DOI:** 10.3390/ma18225124

**Published:** 2025-11-11

**Authors:** Liuchang Zhang, Yue Meng, Shuliang Cheng, Shuo Zhang, Yajun Xin, Yongtao Sun, Qingxin Zhao

**Affiliations:** 1 State Key Laboratory of Metastable Materials Science and Technology, Yanshan University, Qinhuangdao 066004, China; yanshanzlc@outlook.com (L.Z.); xinyajun@ysu.edu.cn (Y.X.); zhaoqx2002@163.com (Q.Z.); 2 Hebei Key Laboratory of Mechanical Reliability for Heavy Equipment and Large Structure, Yanshan University, Qinhuangdao 066004, China; mengy0222@163.com (Y.M.); 18348590526@163.com (S.Z.); 3 Hebei Key Laboratory of Green Construction and Intelligent Maintenance for Civil Engineering, Yanshan University, Qinhuangdao 066004, China; 4 Hebei Province Engineering Research Center for Harmless Synergistic Treatment and Recycling of Municipal Solid Waste, Yanshan University, Qinhuangdao 066004, China; 5 Tianjin Key Laboratory of Nonlinear Dynamics and Chaos Control, Tianjin University, Tianjin 300072, China; 6 Department of Mechanics, Tianjin University, Tianjin 300072, China

**Keywords:** seismic metamaterials, local resonance, auxetic materials, bandgap, seismic isolation

## Abstract

Due to their long wavelengths and low attenuation characteristics, seismic waves pose serious threats to engineering structures, resulting in an urgent need to develop effective vibration mitigation strategies. Locally resonant phononic crystals provide a novel approach to controlling seismic wave propagation, while auxetic materials have attracted considerable attention for their excellent energy absorption capabilities. To achieve broadband low-frequency seismic isolation, this study proposes a seismic metamaterial composed of embedded dual resonators combined with auxetic materials. The bandgap characteristics of the structure are calculated using the finite element method, and the mechanism of bandgap formation is elucidated through vibrational mode analysis. A parametric study is conducted to investigate the influence of mass block substitution on bandgap tunability, and complex band analysis is employed to evaluate seismic wave attenuation within the bandgap range. Furthermore, a graded composite structure is designed, and its seismic isolation performance is validated through frequency- and time-domain simulations. The results show that the proposed composite structure exhibits significant isolation effects within the 2.7–5 Hz bandgap range. Even under excitation with the Chi-Chi earthquake, whose dominant frequency lies outside the bandgap, the peak ground acceleration is reduced by approximately 42%, and the overall acceleration response is effectively suppressed. These findings provide a promising new design strategy for achieving broadband and low-frequency seismic protection in engineering applications.

## 1. Introduction

Earthquakes [1,2,3,4,5], as some of the most destructive natural disasters, pose a serious threat to human life and property due to their sudden onset, wide range of impact, long wavelengths, and high energy that is difficult to attenuate. Statistics show that the frequency of earthquakes is mainly concentrated below 20 Hz. In this frequency range, seismic waves have extremely long wavelengths and strong penetration capabilities, making it difficult for traditional seismic-resistant design methods to achieve effective attenuation. Current seismic protection strategies follow two primary approaches: seismic resistance and seismic isolation. The former focuses on enhancing the stiffness and strength of buildings to improve their intrinsic resistance to seismic waves. However, the effectiveness of this approach is limited, it is often accompanied by irreversible structural damage, and it typically offers a one-time protective effect. In contrast, seismic isolation aims to establish a barrier [6,7,8,9,10] between the structure and incoming seismic waves, thereby blocking or weakening the transmission of seismic energy and significantly reducing the structural vibration response. Therefore, the development of efficient seismic isolation technologies is key to improving seismic protection capabilities.

Studies have shown that body waves [11,12,13,14] traveling from deep within the Earth transform into Rayleigh waves upon reaching the free surface. These surface waves [15,16] concentrate energy near the ground and propagate over a wide area, making them the primary cause of structural damage. In recent years, acoustic metamaterials [17,18,19] based on the local resonance mechanism have been widely applied in vibration control and seismic protection due to their ability to generate bandgaps [20,21,22,23] at subwavelength scales, thereby impeding the propagation of elastic waves [24,25,26,27]. Compared with Bragg scattering [28,29,30] which relies on the periodic structure’s geometric scale locally resonant [31,32,33,34] metamaterials utilize a “matrix–spring–mass block” design to create bandgaps at frequencies far below the structure’s characteristic dimension, making them a promising solution for low-frequency seismic isolation. However, most existing studies have mainly focused on single-resonator systems, which are often difficult to achieve a broad bandgap simultaneously.

With the advancement of related research, scholars have begun to explore the practical application of metamaterials in seismic engineering. Brûlé et al. [35] first introduced the concept of periodicity into seismic protection and theoretically and experimentally demonstrated that embedding periodic cylindrical holes in soil can effectively reflect Rayleigh wave energy. Subsequently, Colombi et al. [36,37] proposed the concept of “seismic metamaterial forests,” where tree arrays induce rainbow effects and wave energy conversion, enabling the attenuation of Rayleigh waves and their conversion into body waves reflected back into the ground. Zeng et al. [38,39] proposed a nested structure strategy for two-dimensional metamaterials to realize multiple low-frequency bandgaps and significantly enhance seismic wave attenuation. Wu et al. [40] introduced cork infill and internal cavity designs to broaden the bandgap frequency range without altering the structural geometry. Zeng et al. [41] were the first to incorporate the inertia amplification mechanism into seismic metamaterials, achieving low-frequency bandgaps with relatively small masses, thereby opening a new avenue for lightweight low-frequency isolation. Luo et al. [42] designed a rigid-boundary meta-barrier that achieves a zero-frequency bandgap in the 0–2 Hz range, providing a new pathway for suppressing extremely low-frequency seismic waves. Meanwhile, auxetic materials [43] have also received increasing attention in vibration attenuation applications due to their unique lateral expansion behavior and excellent energy absorption capacity. Research by Huang et al. [44] indicated that integrating auxetic materials into seismic metamaterials can significantly enhance damping and energy dissipation, facilitating the formation of wider and lower-frequency bandgaps. Despite the ongoing progress in metamaterial-based seismic protection, several limitations remain: single-resonator structures struggle to simultaneously achieve wide bandgaps and low starting frequencies; research on seismic metamaterials incorporating auxetic materials remains limited; and the energy dissipation capacity outside the bandgap frequencies has yet to be validated.

To address these issues, this study proposes a novel embedded seismic metamaterial structure that couples dual resonators with auxetic materials. The research focuses on the following aspects: establishing a dual-resonator seismic metamaterial model and computing its dispersion curves and mode shapes using the finite element method to analyze the bandgap formation mechanisms; conducting a parametric study to investigate the impact of resonator material substitution on bandgap tunability; introducing complex band theory to evaluate seismic wave attenuation within the bandgap frequency range; and developing a gradient composite structure (SM-C) to simulate seismic wave propagation in semi-infinite space under both frequency-domain and time-domain conditions, thereby validating its isolation performance under real seismic loading.

## 2. Model and Theoretical Framework

### 2.1. Model Parameters

**Figure 1:**
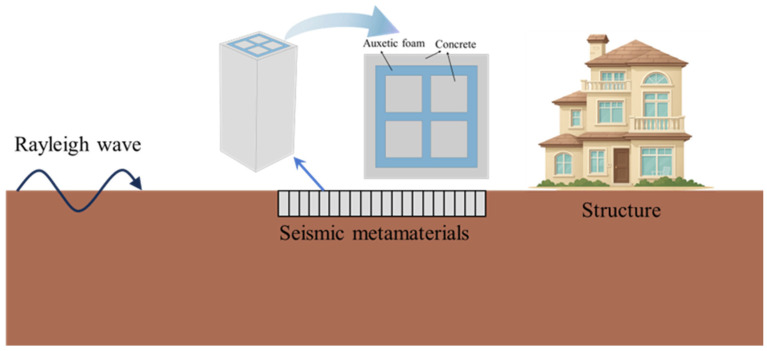
Schematic diagram of the seismic metamaterial structure layout.

Seismic waves, due to their low frequency and long wavelength characteristics, possess strong penetration capabilities and are highly resistant to attenuation. The development of locally resonant phononic crystals has offered a novel approach to subwavelength attenuation of seismic waves, enabling the formation of bandgaps at structural scales much smaller than the wavelength, and thus effectively suppressing low-frequency elastic wave propagation. Leveraging the excellent vibration attenuation performance of the local resonance mechanism, this study proposes a composite resonator-type seismic metamaterial structure and incorporates auxetic materials to investigate its isolation performance. The designed seismic metamaterial is illustrated in Figure 1. The structure is embedded in the soil surrounding a building in a circular configuration, forming a protective barrier that shields the building from Rayleigh waves, which are generated when seismic body waves reach the ground surface. By obstructing the energy transmission path of seismic waves, structural protection of the building can be effectively achieved.

**Figure 2:**
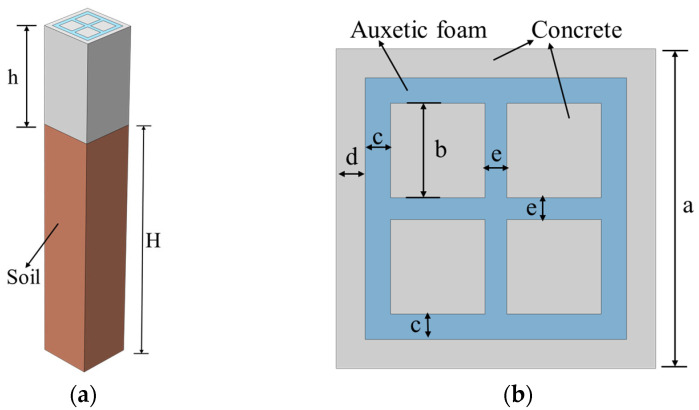
Schematic of the seismic metamaterial structure: (**a**) Three-dimensional diagram; (**b**) Top diagram.

To accurately evaluate the vibration isolation performance of the structure, the model includes not only the dual-resonator seismic metamaterial but also the underlying soil region. This setup comprehensively simulates the boundary constraint effects introduced by actual embedded conditions and their influence on seismic wave attenuation. The composite model more realistically reflects the dynamic response of the metamaterial in a buried environment. The geometric configuration of the seismic metamaterial is shown in Figure 2, and the corresponding structural parameters are listed in Table 1.

Considering practical engineering applications, conventional concrete is selected as the base material of the metamaterial. Within the structure, three horizontal and three vertical staggered grooves are designed and filled with auxetic foam material exhibiting a negative Poisson’s ratio, thereby endowing the structure with excellent energy absorption capability. Four small mass blocks are embedded inside the structure, constructed using a nested combination of concrete and iron to form a dual-resonator system. The detailed material parameters are listed in Table 2.

### 2.2. Theoretical Basis

When the metamaterial structure is subjected to a point disturbance in space, due to the continuity of the structural material medium, the surrounding medium near that point also undergoes spatial displacement in response to the excitation. This propagation of disturbance corresponds to the propagation of elastic waves within the structure. By substituting the geometric and physical equations into the momentum balance equation, the elastic wave equation can be derived as follows:

(1) $$\rho \frac{\partial^{2}u}{\partial t^{2}}=(\lambda +\mu )\nabla (\nabla \cdot u)+\mu \nabla^{2}u$$

where ρ denotes the material density, u is the displacement vector, λ and μ are the Lamé constants. The propagation equation of elastic waves in a unit structure can be expressed in matrix form as:

(2) $$(K-\omega^{2}M)u=F$$

In Equation (2), ***K*** and ***M*** represent the stiffness matrix and mass matrix of the structure, respectively. Since the structure exhibits periodic boundary conditions, the Bloch theorem is introduced to handle the boundary conditions of a single structural unit arranged periodically. The wave function is expressed as:

(3) $$u(r+a)=e^{-ik\cdot a}u(r)$$

By scanning the boundaries of the irreducible Brillouin zone using Equations (2) and (3), the hybrid modes under bulk and surface wave conditions can be obtained.

COMSOL 6.3 solid mechanics is used to calculate the dispersion curves of the metamaterial by scanning the wave vector k to solve for the eigenfrequencies ω. This method only yields the real part of the band structure, which indicates the frequency ranges of the bandgaps. To investigate the energy dissipation characteristics of Rayleigh waves within the bandgap frequency range, and to verify the accuracy of the band structure obtained by COMSOL 6.3, this study employs an inverse approach by scanning the frequency ω to solve for the complex wave vector k. This allows obtaining the imaginary part of the band structure, thereby confirming the accuracy of the FEM simulation results. The complex band structure is computed through the Partial Differential Equation (PDE) module in COMSOL 6.3 by solving the coefficients of the differential equations. The governing equation is as follows:

(4) $$\Lambda^{2}e_{a}u-\Lambda d_{a}u+\nabla \cdot (-c\nabla u-\alpha u+\gamma )+\beta \cdot \nabla u+au=f$$

In the above equation, the eigenvalue is denoted by Λ=−ik; $e_{a}$ is the mass coefficient; $d_{a}$ is the damping coefficient; c is the diffusion coefficient; α represents the convection coefficient for the conserved flux; γ is the absorption coefficient; β denotes the source term for the conserved flux; a is the convection coefficient; and f represents the source term.

Let the wave vector be k=k(cosθ,sinθ,0). By incorporating the Bloch theorem into the elastic wave equation (Equation (2)) and comparing it with Equation (4), the equation coefficients can be determined. This study only verifies the band structure along the Γ to M direction; therefore, θ is set to zero to satisfy wave propagation along the x axis. The detailed coefficients in Equation (4) are as follows:

(5) $$\alpha =\left[\begin{array}{ccc} \left(\begin{array}{c} \Lambda (\lambda +2\mu )\\ 0\\ 0 \end{array}\right) & \left(\begin{array}{c} 0\\ \Lambda \mu \\ 0 \end{array}\right) & \left(\begin{array}{c} 0\\ 0\\ \Lambda \mu \end{array}\right)\\ \left(\begin{array}{c} 0\\ \Lambda \lambda \\ 0 \end{array}\right) & \left(\begin{array}{c} \Lambda \mu \\ 0\\ 0 \end{array}\right) & \left(\begin{array}{c} 0\\ 0\\ 0 \end{array}\right)\\ \left(\begin{array}{c} 0\\ 0\\ \Lambda \lambda \end{array}\right) & \left(\begin{array}{c} 0\\ 0\\ 0 \end{array}\right) & \left(\begin{array}{c} \Lambda \mu \\ 0\\ 0 \end{array}\right) \end{array}\right]$$

(6) $$\beta =\left[\begin{array}{ccc} \left(\begin{array}{c} -\Lambda (\lambda +2\mu )\\ 0\\ 0 \end{array}\right) & \left(\begin{array}{c} 0\\ -\Lambda \lambda \\ 0 \end{array}\right) & \left(\begin{array}{c} 0\\ 0\\ -\Lambda \lambda \end{array}\right)\\ \left(\begin{array}{c} 0\\ -\Lambda \mu \\ 0 \end{array}\right) & \left(\begin{array}{c} -\Lambda \mu \\ 0\\ 0 \end{array}\right) & \left(\begin{array}{c} 0\\ 0\\ 0 \end{array}\right)\\ \left(\begin{array}{c} 0\\ 0\\ -\Lambda \mu \end{array}\right) & \left(\begin{array}{c} 0\\ 0\\ 0 \end{array}\right) & \left(\begin{array}{c} -\Lambda \mu \\ 0\\ 0 \end{array}\right) \end{array}\right]$$

(7) $$e_{a}=\left(\begin{array}{ccc} -(\lambda +2\mu ) & 0 & 0\\ 0 & -\mu & 0\\ 0 & 0 & -\mu \end{array}\right)$$

(8) $$c=\left[\begin{array}{ccc} \left(\begin{array}{ccc} \lambda +2\mu & 0 & 0\\ 0 & \mu & 0\\ 0 & 0 & \mu \end{array}\right) & \left(\begin{array}{ccc} 0 & \lambda & 0\\ \mu & 0 & 0\\ 0 & 0 & 0 \end{array}\right) & \left(\begin{array}{ccc} 0 & 0 & \lambda \\ 0 & 0 & 0\\ \mu & 0 & 0 \end{array}\right)\\ \left(\begin{array}{ccc} 0 & \mu & 0\\ \lambda & 0 & 0\\ 0 & 0 & 0 \end{array}\right) & \left(\begin{array}{ccc} \mu & 0 & 0\\ 0 & \lambda +2\mu & 0\\ 0 & 0 & \mu \end{array}\right) & \left(\begin{array}{ccc} 0 & 0 & 0\\ 0 & 0 & \lambda \\ 0 & \mu & 0 \end{array}\right)\\ \left(\begin{array}{ccc} 0 & 0 & \mu \\ 0 & 0 & 0\\ \lambda & 0 & 0 \end{array}\right) & \left(\begin{array}{ccc} 0 & 0 & 0\\ 0 & 0 & \mu \\ 0 & \lambda & 0 \end{array}\right) & \left(\begin{array}{ccc} \mu & 0 & 0\\ 0 & \mu & 0\\ 0 & 0 & \lambda +2\mu \end{array}\right) \end{array}\right]$$

(9) $$a=\left(\begin{array}{ccc} -\rho \omega^{2} & 0 & 0\\ 0 & -\rho \omega^{2} & 0\\ 0 & 0 & -\rho \omega^{2} \end{array}\right)$$

All other coefficients in Equation (4) are set to zero. In the PDE module, periodic boundary conditions are defined using u(r+a)=u(r) to replace the Bloch periodic continuity condition.

## 3. Band Structure Analysis

In this section, the single-material concrete resonator structure SM-0 (see Figure 2b) is first modeled and analyzed. Finite element simulations are conducted using COMSOL 6.3 to compute the dispersion relations of the structure, which include hybrid modes of bulk and surface waves. Since surface waves (primarily Rayleigh waves) play a dominant role in causing damage to surface structures during seismic events, the analysis in this study focuses specifically on these wave components.

**Figure 3:**
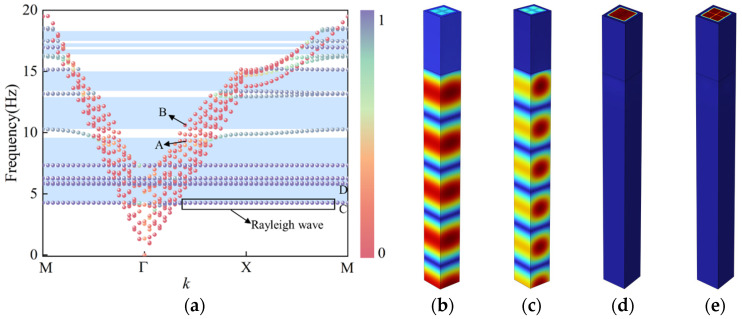
Structure of the monomaterial concrete resonator: (**a**) Dispersion curve, (**b**) Body wave vibration mode A, (**c**) Body wave vibration mode B, (**d**) Surface wave vibration mode C, (**e**) Surface wave vibration mode D.

Figure 3a shows the dispersion curves of the hybrid modes for the structure. Two points within the acoustic cone, A and B, are selected, and their corresponding vibration modes are illustrated in Figure 3b,c. It can be observed that the vibrational energy in this frequency range is primarily concentrated in the lower part of the structure, that is, the soil region, indicating that these modes are mainly associated with seismic body waves, with minimal displacement excited within the metamaterial region. In contrast, two points outside the acoustic cone, C and D, are selected, corresponding to flat dispersion curves. Their associated vibration modes, shown in Figure 3d,e, exhibit vibrations primarily concentrated at the top of the structure, with significant displacement observed particularly in the upper part of the metamaterial. This indicates that these modes are dominated by surface waves.

Given that seismic body waves cause relatively less damage to surface structures, this study distinguishes between body wave modes and surface wave modes based on the distribution of modal vibrational energy [45,46,47]. The classification of the dispersion curves is performed accordingly. The specific criterion for mode identification is given as follows:

(10) $$\beta =\frac{{∭}_{h}({\left|u_{x}\right|}^{2}+{\left|u_{y}\right|}^{2}+{\left|u_{z}\right|}^{2})dV}{{∭}_{h+H}({\left|u_{x}\right|}^{2}+{\left|u_{y}\right|}^{2}+{\left|u_{z}\right|}^{2})dV}$$

The polarization factor β is employed to quantify the distribution characteristics of modal vibrational energy on the structural surface. A value of β approaching 1 indicates that the displacement of the mode is primarily concentrated on the surface, whereas a lower value suggests that the vibration mainly occurs within the interior of the structure. In this study, frequency points with high polarization factor β>0.9 are identified as surface wave modes, whose vibrational characteristics effectively represent the propagation behavior of Rayleigh waves.

Figure 3a presents the distribution of the calculated polarization factor. It can be observed that the modes within the acoustic cone are all body wave modes, with vibrational energy primarily concentrated in the lower part of the structure, and negligible energy transmitted to the surface. Therefore, the subsequent analysis in this study focuses solely on surface wave modes exhibiting significant surface vibration characteristics. After introducing the auxetic foam into the structure, multiple broadband bandgaps below 10 Hz are effectively generated, with the lowest bandgap starting at 4.3 Hz.

**Figure 4:**
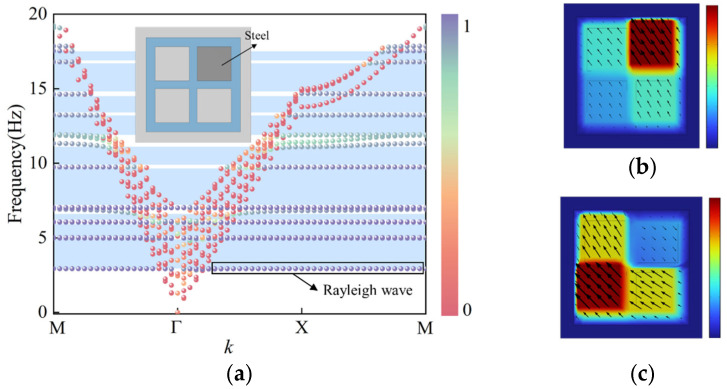
Structure with an embedded iron block: (**a**) Dispersion curves, (**b**) vibration mode at the lower edge of the first bandgap, (**c**) vibration mode at the lower edge of the second bandgap.

The seismic wave energy is primarily concentrated below 5 Hz. To achieve the formation of lower-frequency bandgaps, a dual-resonator design is introduced based on the original structure, in which the small concrete block located at the upper right corner is replaced with an iron block, as shown in Figure 4a. The introduction of iron blocks reduces the lower bound of the initial bandgap to 2.9 Hz, representing a 1.4 Hz decrease compared to the single-resonator structure without iron blocks. Additionally, the bandgap width below 10 Hz is increased by 1 Hz, and the number of bandgaps within 20 Hz rises significantly, indicating that the dual-resonator design offers notable advantages in low-frequency seismic isolation performance.

The coupling effect of the dual-resonator design reduces the fluctuation of the surface mode dispersion curves, rendering the curves nearly flat and significantly broadening the low-frequency bandgap. As shown in Figure 4b, the vibration mode at the lower edge of the first bandgap is primarily concentrated in the iron blocks, which have a lower natural frequency. These blocks exhibit strong resonant behavior, while the base material responds only weakly. This indicates that the structure effectively confines seismic wave energy within the resonant units, forming a locally resonant bandgap. Due to the flatness of the dispersion curve at this frequency, the corresponding group velocity approaches zero, implying that seismic waves cannot propagate at this frequency, thereby achieving the desired isolation effect.

The vibration mode at the lower edge of the second bandgap is shown in Figure 4c. At this frequency, the structural vibration is primarily governed by the concrete blocks, which possess higher natural frequencies. These blocks effectively capture seismic wave energy within the resonators, thereby initiating the second bandgap. Through the coupling mechanism between different materials, the dual-resonator configuration facilitates a synergistic effect that broadens the low-frequency bandgaps and enhances the seismic isolation performance of the structure.

**Figure 5:**
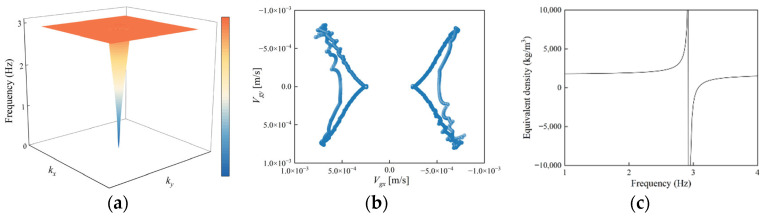
Structure with an embedded iron block: (**a**) First dispersion surface, (**b**) group velocity at 2.9 Hz, (**c**) effective negative density.

To further investigate the relationship between bandgap formation and energy flow, the group velocity is introduced as a parameter characterizing energy propagation. The magnitude and direction of the group velocity correspond to the rate and direction of energy transmission, respectively. Physically, it is defined as the gradient of frequency with respect to the wave vector, that is, $v_{g}=(\partial \omega /\partial k_{x})i+(\partial \omega /\partial k_{y})j$, effectively reflecting the ability of waves to propagate within the structure. Using the global boundary method, the formula for extracting effective negative density is expressed as: $\rho_{eff}=F/A$, where F denotes the reaction force on the structure boundary, and A is the boundary acceleration. Figure 5a presents the first dispersion surface of the SM-1 structure, which exhibits typical characteristics of a locally resonant metamaterial. A pronounced flat region appears on the dispersion surface, corresponding to surface wave modes, whereas the conical ascending part indicates the bulk wave dominant region. The flat dispersion region implies wave localization and inhibited propagation.

Figure 5b illustrates the propagation characteristics at this frequency by depicting the distribution of the group velocity vectors at the lower boundary of the band gap. The results indicate that the magnitude of the group velocity is extremely low, approaching zero, and is significantly lower than the propagation speed of conventional seismic waves. Combined with the vibration mode shown in Figure 4b and the negative effective density presented in Figure 5c at the band gap lower boundary, it can be concluded that strong local resonance occurs in the iron block at this frequency. This local resonance confines the wave energy within a localized region, causing the wave to transform from a traveling wave to a standing wave, thereby preventing energy transmission to the surroundings and resulting in the formation of the band gap.

Two iron blocks were embedded simultaneously at the diagonal positions of the structure, designated as SM-2, and its dispersion characteristics were computed. The results indicate that the lower boundary of the initial band gap further decreased to 2.7 Hz, representing a 0.2 Hz reduction compared to the structure with a single iron block. Compared with the structure embedding only one iron block, the double iron block structure exhibits a stronger resonance effect, not only merging the low-frequency band gaps but also opening a new band gap at an even lower frequency, significantly enhancing the isolation capability against low-frequency seismic waves.

**Figure 6:**
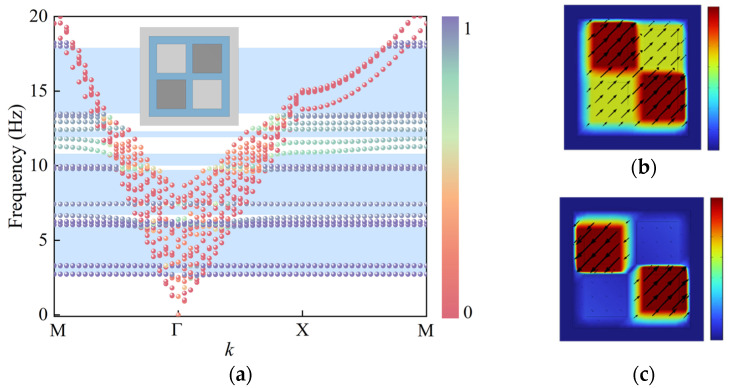
Structure with two embedded iron blocks: (**a**) Dispersion curves (**b**) vibration mode at the lower boundary of the initial band gap, (**c**) vibration mode at the lower boundary of the second band gap.

Figure 6b illustrates the typical vibration mode at the lower boundary of the first band gap: the two iron blocks arranged diagonally exhibit in-phase oscillations. The strong resonance confines the energy within the resonators, preventing its propagation to the surroundings and thereby forming a pronounced local resonance band gap. The vibration mode at the lower boundary of the second band gap (Figure 6c) shows the two iron blocks vibrating out of phase. This mode similarly traps seismic wave energy within the resonators to open the band gap, further broadening the band gap range.

In summary, the resonator structure with two iron blocks arranged diagonally achieves a broader band gap width and a lower band gap starting frequency through the synergistic effects of multiple resonance modes, providing an effective approach to realizing superior ultra-low-frequency seismic isolation performance.

**Figure 7:**
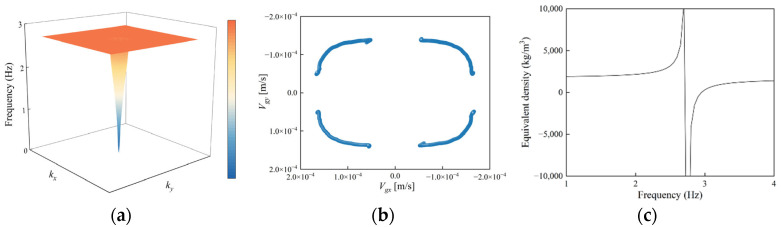
Structure with two embedded iron blocks: (**a**) First dispersion surface, (**b**) group velocity at 2.7 Hz, (**c**) negative effective mass density.

Figure 7a shows the first dispersion surface of the SM-2 structure. It can be observed that the structure exhibits pronounced local resonance characteristics in the low-frequency region. After introducing the high-density mass blocks, although the overall shape of the dispersion surface changes little, its thickness is significantly compressed, and the frequency at the upper dispersion surface shifts downward to 2.7 Hz, effectively lowering the starting frequency of the band gap. Figure 7b indicates that the magnitude of the group velocity approaches zero at the lower boundary of the band gap, further confirming the presence of the local resonance mechanism. Combined with the vibration mode at 2.7 Hz shown in Figure 6b, significant vibration occurs in the embedded iron block regions, and the dual iron blocks enhance the structure’s ability to capture energy. Figure 7c further reveals negative effective mass density near 2.7 Hz. This inertial reversal response strongly suppresses elastic wave propagation, confining energy within a localized region and forming local elastic wave modes. Compared to the SM-1 structure, SM-2 demonstrates superior performance in energy regulation.

**Figure 8:**
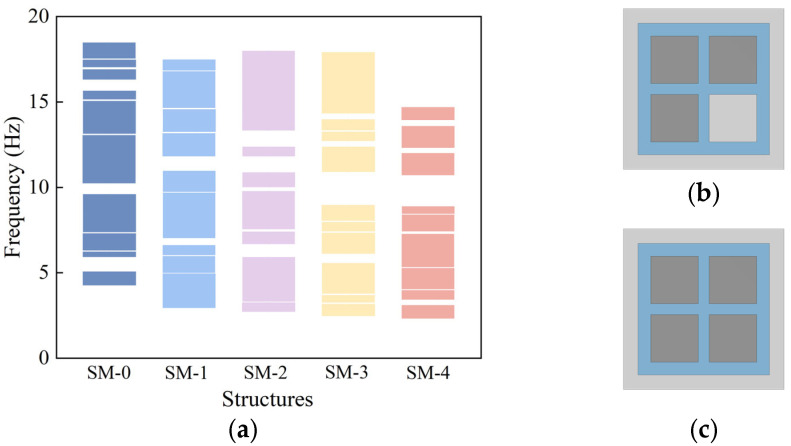
(**a**) Effect of the number of embedded iron blocks on the band gap, (**b**) Structure with three embedded iron blocks, (**c**) Structure with four embedded iron blocks.

**Figure A1:**
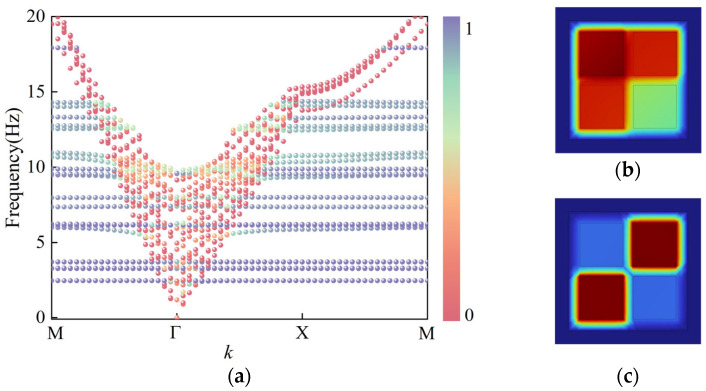
Structure with three embedded iron blocks: (**a**) Dispersion curves (**b**) vibration mode at the lower boundary of the initial band gap, (**c**) vibration mode at the lower boundary of the second band gap.

**Figure A2:**
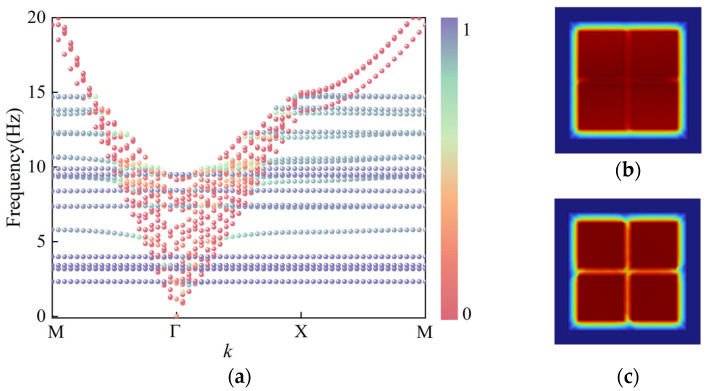
Structure with four embedded iron blocks: (**a**) Dispersion curves (**b**) vibration mode at the lower boundary of the initial band gap, (**c**) vibration mode at the lower boundary of the second band gap.

To further investigate the effect of the number of resonator iron blocks on the bandgap characteristics, three and four iron blocks were embedded in the structure, referred to as SM-3 and SM-4 (The dispersion curves and vibration modes are shown in Appendix A Figure A1 and Figure A2), respectively. The structural schematics are shown in Figure 8b,c, while the corresponding dispersion curves and bandgap distributions are presented in Figure 8a (The specific frequency range of the band gap is provided in Appendix A Table A1). Comparison with the previous structures reveals that, as the number of embedded iron blocks increases, the lower boundary of the initial bandgap gradually decreases, indicating that increasing the number of resonators helps to extend the bandgap to lower frequency ranges.

However, when all four mass blocks in the structure were replaced with high-density iron blocks, although the lower boundary of the initial bandgap further decreased, resulting in the formation of the lowest frequency bandgap, the overall bandgap coverage significantly narrowed. While the use of high-density materials reduces the structure’s natural frequency to open low-frequency bandgaps, it simultaneously weakens the structure’s ability to control mid-to-high frequency waves. Increasing the number of resonators fully replaced by high-density materials does not always positively impact bandgap performance, leading to a reduced bandgap width and thus lowering the seismic isolation frequency range. The dual-resonator design, which combines two high-density iron blocks with two low-density concrete blocks, effectively opens low-frequency bandgaps while maintaining a relatively wide bandgap coverage. This achieves a good balance between lowering the starting frequency of the bandgap and expanding the bandgap width, thereby validating the effectiveness and engineering application potential of the dual-resonator design in seismic metamaterial development.

The real band structure calculation results of the structure have been presented earlier. Although the real band structure effectively shows the frequency ranges where waves can propagate within the structure, it appears as a blank in the bandgap regions and cannot reflect the differences in elastic wave energy attenuation capabilities among different bandgaps. Therefore, this study further employs the PDE method to calculate the complex band structure [48,49,50], analyzing the attenuation characteristics of elastic waves within the structure.

**Figure 9:**
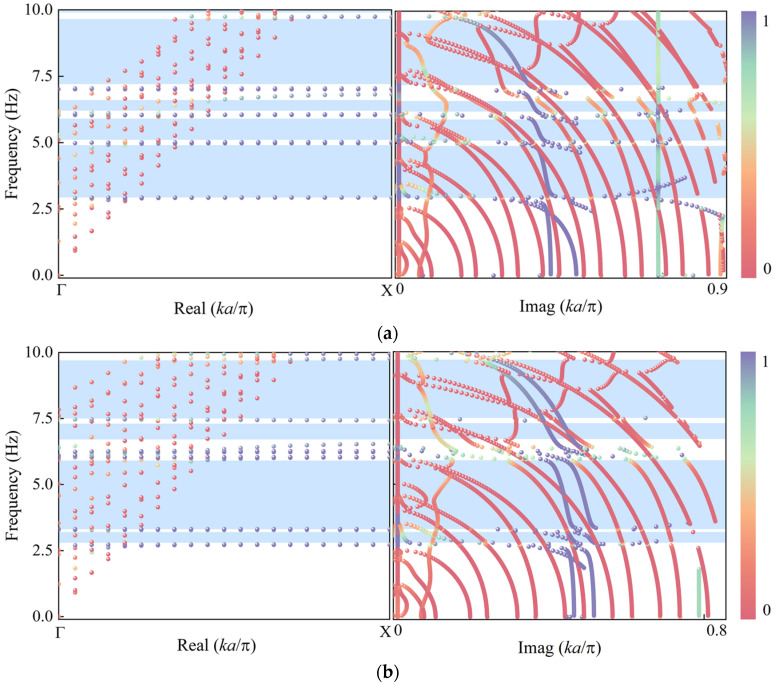
Complex Band Gap: (**a**) structure with an embedded iron block; (**b**) structure with two embedded iron blocks.

As shown in Figure 9a, the calculated complex band structure highly agrees with the previously presented real band structure. Within the real domain of the wave vector Real(kπ/a)>0, Imag(kπ/a)=0, the elastic waves propagate in the metamaterial without attenuation, whereas in the complex domain Real(kπ/a)=0, Imag(kπ/a)>0, the elastic waves exhibit exponential decay within the structure, corresponding to the attenuation modes inside the bandgap. The figure clearly illustrates that the complex band structure displays propagating modes in the passband regions and transitions to evanescent modes in the bandgap regions, effectively reflecting the transition from traveling waves to decaying waves.

Further analysis of the characteristics of the complex band curves reveals that the imaginary part forms cusps at the bandgap boundaries, and the overall curve shape exhibits asymmetry. This indicates that the bandgaps are predominantly governed by the local resonance mechanism, distinctly different from the highly symmetric shape of Bragg scattering-induced complex bands. Figure 9a shows that the four bandgaps formed within the structure correspond to pronounced energy attenuation regions, with the bandgaps below 7 Hz exhibiting larger imaginary components of the wave vector, indicating stronger elastic wave attenuation by the structure in the low-frequency range. Figure 9b illustrates the changes in the complex band structure after introducing two iron blocks into the system. The results indicate that the structure forms a wider low-frequency bandgap in the 3.3 Hz to 6 Hz range and achieves bandgap merging within the 6 Hz to 9.6 Hz interval, thereby broadening the overall seismic isolation frequency band. Additionally, the imaginary part of the wave vector remains significant in both frequency ranges, reflecting the enhanced elastic wave attenuation capability of the structure at low frequencies.

## 4. Structural Attenuation Performance

**Figure 10:**
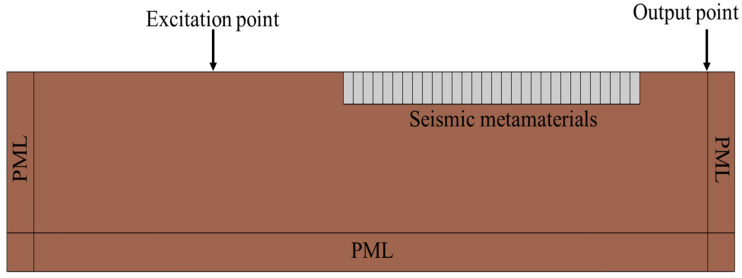
Semi-infinite Space Model for Frequency and Time Domain Analysis.

Based on the completed calculation of the seismic metamaterial dispersion curves, this study further verifies the structure’s attenuation capability against seismic waves at specific frequencies by employing both frequency-domain and transient analyses. Considering that actual seismic waves propagate in a semi-infinite space, a semi-infinite space model is constructed to simulate realistic conditions, as shown in Figure 10. In the frequency-domain analysis, a perfectly matched layer (PML) is applied around the soil boundaries to effectively absorb seismic waves reaching the boundaries and to avoid reflection interference in the results. In the transient analysis, low-reflecting boundary conditions are imposed to suppress wave reflections at the boundaries.

Since Rayleigh waves are the most destructive component of seismic waves to building structures, this study applies excitation on the model surface to simulate the propagation of Rayleigh waves along the surface. Receiving points are arranged behind the seismic metamaterial structure. By comparing the vibration responses at the receiving points with and without the seismic metamaterial structure, the differences can be analyzed to further verify the seismic isolation effectiveness of the structure under actual seismic wave excitation.

### 4.1. Frequency Domain Analysis

To facilitate the description of seismic wave attenuation after incorporating the metamaterial structure, a dimensionless function is defined to characterize the transmission loss of seismic waves by the seismic metamaterial:

(11) $$TL=20\log_{10}(\frac{u_{with}}{u_{without}})$$

where $u_{with}$ and $u_{without}$ represent the displacement responses at the output point with and without the seismic metamaterial structure, respectively.

**Figure 11:**
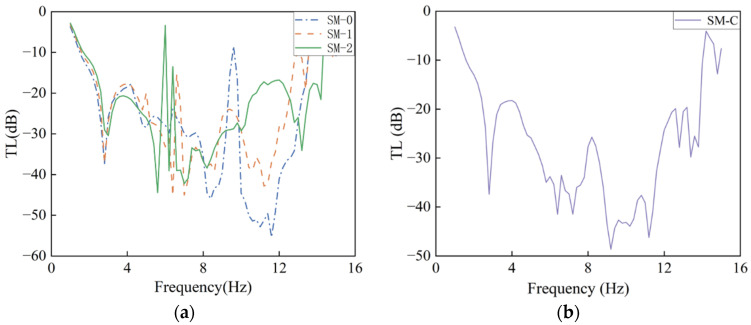
Vibration transmission loss: (**a**) single-unit structure; (**b**) composite structure.

A vertical displacement excitation of 0.1 m was applied at the excitation end to compute the frequency-domain responses of the structures without embedded iron blocks (SM-0), with one iron block (SM-1), and with two iron blocks (SM-2), as shown in Figure 11a. As the number of embedded iron blocks increases, the peak attenuation frequencies gradually shift toward lower frequencies, indicating that increasing the number of resonators enhances the structure’s attenuation capacity for low-frequency seismic waves. Although the SM-2 structure demonstrates excellent attenuation performance around 5 Hz, its attenuation effect at 6 Hz is relatively weak, which is consistent with the band gap distribution shown in Figure 6a. In contrast, the SM-1 structure exhibits significant isolation performance at 6 Hz. Combined with the band gap distribution in Figure 8a, it can be observed that the band gaps of SM-0, SM-1, and SM-2 complement each other and collectively cover the target frequency range of 2.7–15 Hz, demonstrating the potential for collaborative wideband seismic isolation.

Based on the above analysis, a composite structure, denoted as SM-C, was constructed by sequentially arranging 10 units each of SM-0, SM-1, and SM-2. The transmission spectrum of SM-C is shown in Figure 11b. The SM-C structure exhibits significant wave attenuation performance in the frequency range of 2.7–10 Hz. Compared to the reference model without seismic metamaterials, the vibration response in the target frequency band is reduced by more than one order of magnitude, verifying the excellent performance of the composite structure in low-frequency broadband vibration isolation. This further confirms the effectiveness and practical value of the dual-resonator design in engineering applications of seismic metamaterials.

**Figure 12:**
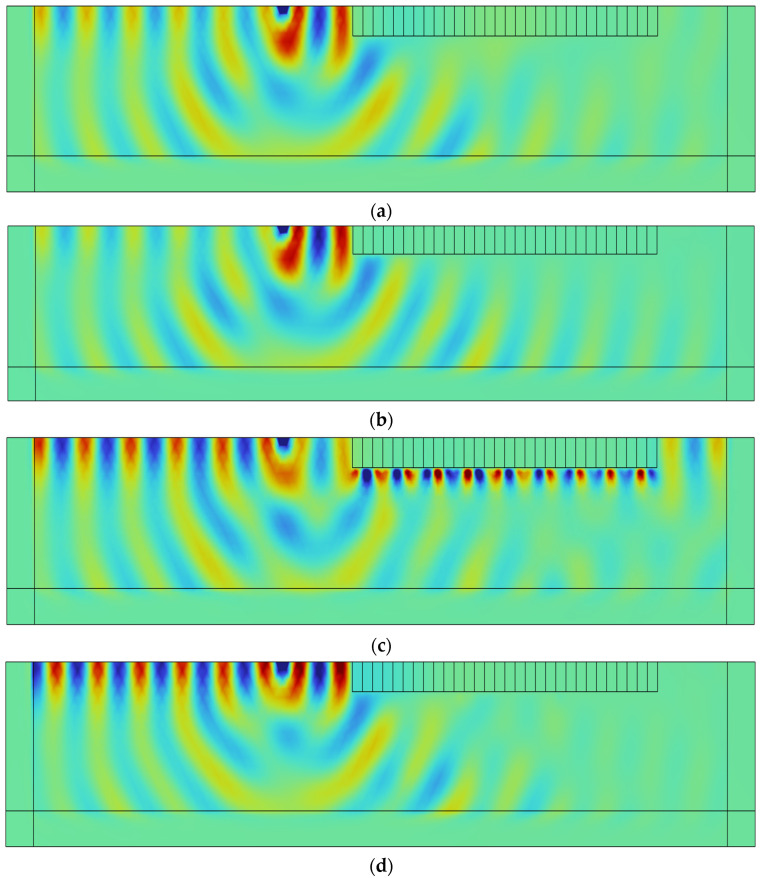
Displacement of the semi-infinite space model at 6 Hz: (**a**) SM-0, (**b**) SM-1, (**c**) SM-2, and (**d**) SM-C.

As shown in Figure 12, at the frequency of 6 Hz, the SM-2 structure exhibits a weak suppression effect on Rayleigh waves. The waves propagate through the seismic metamaterial region with negligible attenuation, indicating limited isolation performance at this frequency. In contrast, the SM-0 and SM-1 structures demonstrate significant attenuation of Rayleigh waves, effectively preventing wave propagation into the interior of the structure. The displacement field results are highly consistent with the previously obtained transmission spectra, further confirming the correlation between the bandgap mechanism and the ability to block seismic wave energy.

In addition, Figure 12d shows the displacement response distribution of the composite structure SM-C under 6 Hz excitation. The results indicate that the Rayleigh wave energy is effectively isolated within the metamaterial region, and its propagation path is significantly interrupted, demonstrating the superior Rayleigh wave suppression capability of the SM-C structure at this frequency. This finding further confirms the application potential of the combined dual-resonator metamaterial structure in seismic protection.

### 4.2. Time-Domain Analysis

Although the above frequency-domain analysis effectively reveals the band gap characteristics and transmission behavior of the seismic metamaterial structure, the applied excitation is periodic and idealized, making it difficult to accurately reflect the dynamic response of the metamaterial under actual seismic wave conditions. Therefore, to comprehensively evaluate the seismic isolation performance of the composite structure SM-C under seismic loading, this study further introduces time-domain analysis to investigate its response under both synthetic seismic waves and real earthquake time-history excitations.

**Figure 13:**
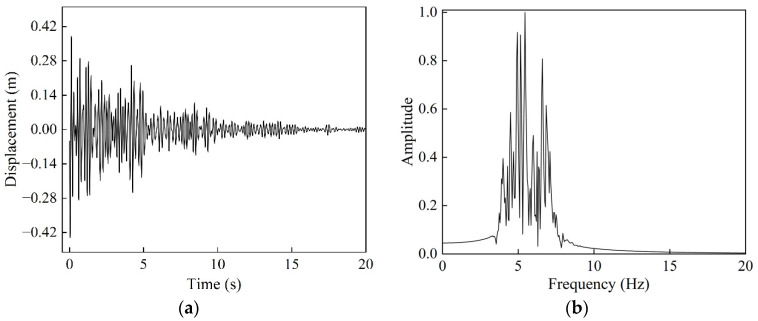
(**a**) Displacement time history curve; (**b**) frequency spectrum.

Firstly, to match the vibration mitigation frequency characteristics of the SM-C structure, a synthetic seismic displacement time-history curve consistent with its band gap range was constructed, with the introduction of a damping factor $e^{-\alpha t}$ to better approximate the dynamic characteristics of actual seismic excitation. The synthetic time-history curve is shown in Figure 13a. Fourier transform and normalization of this time-history yielded its frequency spectrum distribution, as depicted in Figure 13b. The results indicate that the main frequency components of the excitation signal are concentrated between 4 and 7 Hz, which aligns well with the effective vibration mitigation frequency range demonstrated by the transmission characteristic curve of the SM-C structure in Figure 11b.

**Figure 14:**
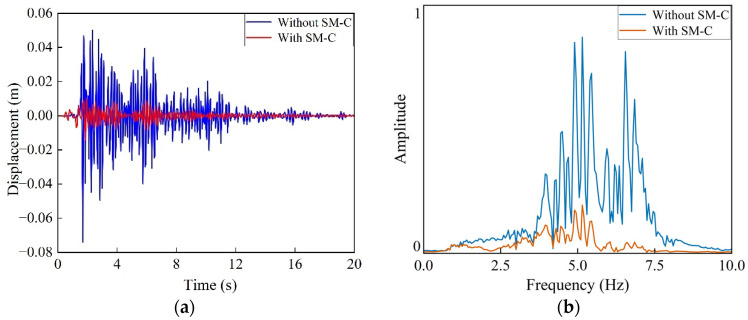
Displacement excitation response of the SM-C structure: (**a**) displacement time history; (**b**) frequency spectrum.

**Figure 15:**
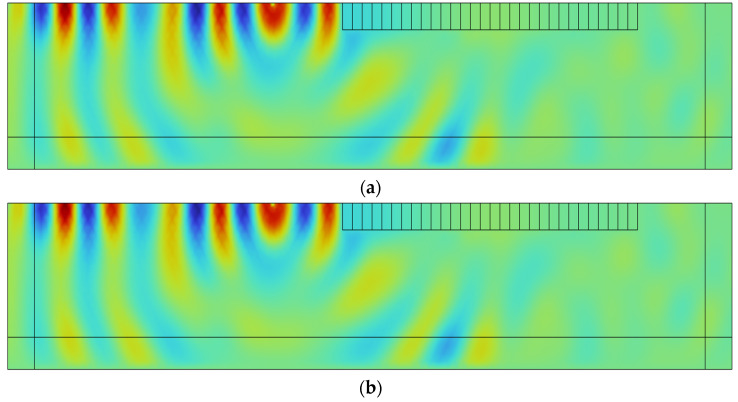
Displacement of the SM-C structure at different times:(**a**) 5 s, (**b**) 8 s.

After applying vertical displacement excitation within the structure’s vibration mitigation frequency range, the resulting structural response is shown in Figure 14a. By comparing the vibration displacement at the observation point before and after introducing the seismic metamaterial structure, a significant reduction in vibration amplitude is observed due to the periodic attenuation effect of the metamaterial, demonstrating excellent vibration mitigation performance. Further Fourier transform analysis of the displacement response reveals that the Rayleigh wave components in the 4–7 Hz frequency band are substantially suppressed after passing through the metamaterial structure. This is consistent with the bandgap frequency range of 4–7 Hz shown in the dispersion curves in Figure 3, Figure 4 and Figure 5, confirming the metamaterial’s effective wave isolation capability within the target frequency band. Displacement contour maps at different time instants (5 s and 8 s) are shown in Figure 15, clearly illustrating that the Rayleigh waves are significantly blocked by the metamaterial region during propagation. The wave energy undergoes substantial attenuation as it passes through this region, further confirming the effective suppression of Rayleigh wave propagation by the SM-C structure.

Since the frequency content of seismic waves is primarily concentrated in the ultra-low frequency range and exhibits significant randomness and uncertainty, these waves are difficult to attenuate naturally during propagation. Therefore, relying solely on idealized excitations or single-frequency analyses cannot comprehensively reflect the actual vibration mitigation performance of the structure. To further evaluate the seismic attenuation effectiveness of the proposed seismic metamaterial structure under realistic earthquake excitations, three representative earthquake acceleration time histories were selected, namely those from the Imperial Valley, El Centro, and Chi-Chi earthquakes. These seismic records were all obtained from the PEER ground motion database.

**Figure 16:**
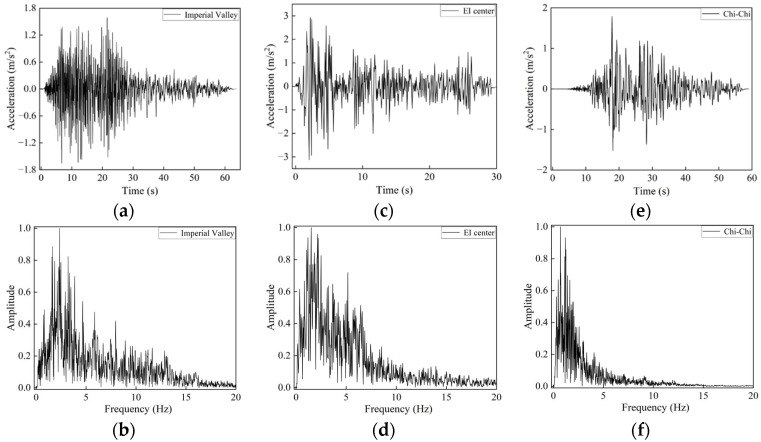
Acceleration time history curve and Frequency spectrum of the seismic wave: (**a**,**b**) Imperial Valley, (**c**,**d**) El Centro, (**e**,**f**) Chi-Chi.

Figure 16 presents the acceleration time histories of the selected seismic waves along with their corresponding spectral characteristics. The spectra reveal that all three seismic records contain abundant low-frequency components below 5 Hz. The frequency contents of the Imperial Valley and El Centro earthquakes closely overlap with the target bandgap frequency range of the proposed structure, providing effective excitation for validating the structure’s seismic isolation performance.

**Figure 17:**
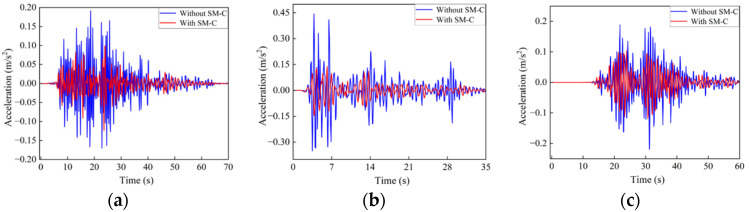
Acceleration excitation response of the SM-C structure: (**a**) Imperial Valley, (**b**) El Centro, (**c**) Chi-Chi.

Figure 17 shows the comparison of acceleration responses before and after the introduction of the seismic metamaterial SM-C under excitation from three sets of real earthquake records. For the Imperial Valley earthquake shown in Figure 17a, the peak acceleration response decreases by approximately 36% after incorporating the SM-C structure. Moreover, the attenuated acceleration response remains stable below 0.05 m/s^2^, especially during the main shock period between 10 and 30 s, where the response amplitude is significantly reduced, indicating that the structure effectively suppresses the propagation of the main seismic energy.

Figure 17b presents the response to the El Centro earthquake, whose energy is primarily distributed within the 2–6 Hz frequency range, closely matching the bandgap frequencies of the SM-C structure. Consequently, the peak acceleration is reduced by 68%, and the overall response amplitude remains below 0.1 m/s^2^ throughout the 35-s seismic event. This demonstrates the excellent vibration suppression capability of the SM-C structure and further confirms its effective seismic attenuation performance within the target frequency band.

Figure 17c presents the acceleration response of the SM-C structure to the Chi-Chi earthquake wave, with the main frequency concentrated between 0 and 2 Hz. As indicated by the previous dispersion curves, there are no surface wave modes in the 0–2 Hz frequency range; only body wave modes exist in this range. Therefore, from the perspective of dispersion, the structure exhibits a zero-frequency band gap in this frequency range. However, due to the long wavelength and strong penetration ability of seismic waves below 2 Hz, the vibration transmission loss calculated for the array structure is relatively small, and it does not reach the vibration attenuation capability typical of band gaps above 3 Hz. As a result, we did not define frequencies below 2 Hz as surface wave band gaps. The selected seismic record is specifically chosen to evaluate the response control capability of the SM-C structure outside the band gap range. The simulation results show that after introducing the SM-C structure, the peak acceleration decreases from 0.19 m/s^2^ to 0.11 m/s^2^, with an overall slight reduction in response amplitude. Although the main frequency of the earthquake exceeds the effective band gap range defined for the structure, the SM-C structure still demonstrates certain seismic isolation and acceleration attenuation capabilities, exhibiting some wave control even at non-ideal frequencies.

## 5. Conclusions

This paper proposes a novel embedded seismic metamaterial structure that integrates dual resonators with materials with a negative Poisson’s ratio. Compared with the structure embedded with a single resonator, the proposed design achieves a broader bandgap coverage and superior seismic isolation performance [51]. By calculating the dispersion curves and analyzing the vibration modes at the lower edge of the bandgap, the mechanism of bandgap formation is clarified. Parametric analysis indicates that gradually replacing the concrete mass blocks with iron blocks facilitates the opening of low-frequency bandgaps, while a complete replacement reduces the overall bandgap coverage. This demonstrates that the dual-resonator configuration not only enables low-frequency seismic isolation but also preserves a wide bandgap characteristic.

On this basis, a graded composite structure, denoted as SM-C, is proposed. When the dominant frequency of the seismic wave falls within the bandgap range, the SM-C structure can significantly attenuate the seismic energy. Even for the Chi-Chi earthquake, whose dominant frequency lies outside the bandgap range, the SM-C structure achieves an approximately 42% reduction in peak ground acceleration, effectively suppressing the overall acceleration response. Furthermore, no seismic wave amplification effects were observed in either the complex band or time-domain analyses. These findings fully demonstrate the remarkable vibration mitigation potential and engineering applicability of the SM-C structure. In future work, the optimization of seismic metamaterial performance will be further explored through machine-learning-assisted design and parameter tuning.

## Figures and Tables

**Table 1:** Geometrical dimensions of the structure.

a (m)	b (m)	c (m)	d (m)	e (m)	h (m)	H (m)
2.2	0.65	0.16	0.2	0.18	5	20

**Table 2:** Material properties of the structural components.

	Density (kg/m^3^)	Young’s Modulus (Pa)	Poisson’s Ratio
Soil	1800	2 × 10^7^	0.3
Concrete	2300	2.5 × 10^10^	0.2
Auxetic foam	120	2.5 × 10^4^	–0.8
Steel	7850	2.05 × 10^11^	0.28

**Figure A3:**
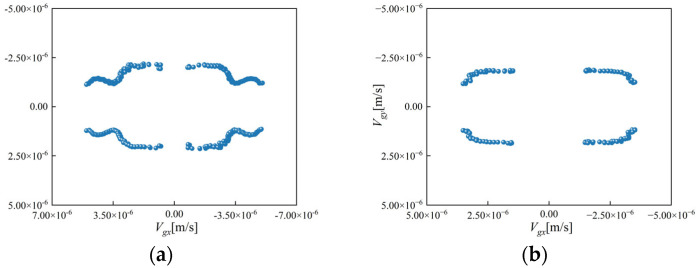
Group velocity at the lower boundary of the initial bandgap: (**a**) SM-3, (**b**) SM-4.

## Data Availability

The original contributions presented in this study are included in the article. Further inquiries can be directed to the corresponding authors.

## Appendix A

**Table A1:** The specific range of the structural bandgap in the manuscript is as follows:.

SM-0	4.3–5.8, 5.89–6.2, 6.4–7.2, 7.4–9.58, 10.3–12.9, 13.4–15, 16.4–16.8, 17–17.3, 17.5–18.3
SM-1	2.9–4.9, 5.1–5.9, 6.2–6.6, 7.2–9.65, 9.9–11.1, 11.9–13.2, 13.4–14.5, 14.8–16.7, 16.9–17.5
SM-2	2.8–3.2,3.3–5.9,6.7–7.3,7.5–9.7,10–10.8,11.9–12.3,13.5–17.9
SM-3	2.46–3.1, 3.2–3.6, 3.7–5.6, 6.1–7.3, 7.4–7.9, 8–9, 10.9–12.4, 12.7–13.2, 13.3–14, 14.3–17.9
SM-4	2.3–3.1, 3.4–3.9, 4–5.2, 5.3–7.3, 7.4–8.3, 8.4–8.9, 10.7–12, 12.3–13.6, 13.9–14.7

When the resonator material is entirely concrete, the structure’s bandgap starts at a frequency of 4.3 Hz. As the number of iron blocks increases, the starting frequency of the bandgap shows an overall downward trend. Additionally, when the resonators contain concrete material, the cutoff frequency of the bandgap remains relatively stable around 18 Hz. When the resonator material is entirely replaced with iron blocks, although the initial bandgap further decreases, a range of frequencies between 15 and 20 Hz exhibits no bandgap coverage. This suggests that the SM-1, SM-2, and SM-3 dual-material resonator embedded structures can provide a broader bandgap coverage, thereby achieving more effective low-frequency vibration isolation.

As shown in Figure A1, the dispersion curves of the SM-4 structure, along with the mode shapes at the lower edges of the initial band gap and the second band gap, are presented. From the mode shape at the lower edge of the initial band gap, it is evident that the three iron blocks within the structure resonate, effectively trapping energy and thereby leading to the opening of the band gap. At the lower edge of the second band gap, the corresponding mode involves the vibration of two iron blocks, which also capture energy through their resonant behavior. This demonstrates that by embedding multiple iron blocks into the structure and combining them in a reasonable manner, multiple resonant frequencies can be formed, thus enabling the opening of multiple band gaps.

As shown in Figure A2, the dispersion curves of the SM-4 structure are presented, along with the vibration modes corresponding to the lower edges of the initial band gap and the second band gap. In the mode at the lower edge of the initial band gap, the four iron blocks within the structure work in synergy, forming a collective resonance that localizes the energy and opens the band gap. In contrast, at the lower edge of the second band gap, the four iron blocks exhibit independent vibrations, with each block relying on its individual resonance to capture energy.

As shown in Figure A3, a comparison of the group velocities at the lower edge of the initial band gap between the SM-3 and SM-4 structures indicates that increasing the number of iron blocks can effectively suppress energy fluctuation within the structure.
